# Supporting Team Reflexivity During the COVID-19 Lockdown: A Qualitative Study of Multi-Vision Groups In-person and Online

**DOI:** 10.3389/fpsyg.2021.719403

**Published:** 2021-08-06

**Authors:** Santa Parrello, Elisabetta Fenizia, Rosa Gentile, Ilaria Iorio, Clara Sartini, Massimiliano Sommantico

**Affiliations:** ^1^Department of Humanities, Section of Psychology and Educational Sciences, University of Naples “Federico II”, Naples, Italy; ^2^Associazione Maestri di Strada ONLUS, Naples, Italy

**Keywords:** socio-educational work, team reflexivity, action-research, COVID-19 lockdown, modified balint group, online group, T-lab text analysis

## Abstract

**Introduction:** The professional self is often hindered by a lack of self-care and poor work-life balance, and cannot be considered an unlimited resource. Given this, the reflexive team is an important organizational tool for protecting workers' well-being. The non-profit organization *Maestri di Strada* (MdS) (“Street Teachers”) conducts action research (AR) in the area of socio-education. The main tool used by the group to protect the well-being of its members is a guided reflexivity group, inspired by the Balint Group and termed the Multi-Vision Group (MG). In March 2020, because of the COVID-19 lockdown, the MdS team had to quickly revamp its working model, and MGs were held online for the first time.

**Aim:** Through qualitative research that takes a longitudinal approach, the aim of this study is to evaluate the efficacy of the MG in supporting the team's reflexivity in this new online format.

**Methods:** This article considers MGs during two different time periods: pre-pandemic (T1) and early pandemic (T2). During T1, the MdS team met 18 times in person, while during T2 the team met 12 times through an online platform (always under the guidance of a psychotherapist). During all sessions in both time periods, a silent observer was present in the meetings, and they subsequently compiled narrative reports. The textual corpora of the reports were submitted for a Thematic Analysis of Elementary Contexts through T-Lab Plus, in order to examine the main content of the groups' discourse.

**Results:** The results (five clusters in T1; and five in T2) show that, during T2, the group devoted considerable time to experiences tied to the pandemic (T21: schools facing the pandemic crisis; T2.2: the pandemic: death, inner worlds, and thought resistance; T2.3: kids' stories involving physical distancing and emotional proximity). The group also came up with innovative educational initiatives that defied the lockdown (T2.4: fieldwork: the delivery of “packages of food for thought”; T2.5: the MdS group: identity and separation). Based on these findings, the MG most likely contributed to the emergence of MdS as a “resilient community,” capable of absorbing the shock of the pandemic and realizing a fast recovery response.

## Introduction

### Professional Self, Well-Being, and Teams

Non-profit organizations are steadily becoming a larger presence in Italy (ISTAT, 2020). However, these organizations face considerable difficulties that impact workers' well-being, such as excessive employee turnover, decreased public funding, delays in contract renewals, and a lack of systematic resources for supervision and the kind of reflection that creates team support (Olivieri, 2019).

The professional self, which is closely related to the personal self, has an intersubjective, narrative, discursive, and reflective structure (Manuti, 2006; Fellenz, 2016). It can be harmed by a lack of self-care and by poor work-life balance (Myers et al., 2020). The professional self cannot be considered an unlimited resource, and because of this, team support is particularly important: new ideas and innovation emerge *between* rather than *within* people, thereby emphasizing the importance of the social and collective context (Barak et al., 2010).

Initial professional training based on knowledge and skills is not enough to support those who work in challenging environments: “on the one hand, knowledge will not just be out of date, but will always be insufficient to describe the novel and unstable situations that present themselves; on the other hand, skills are always addressed to known situations, and cannot be addressed to unforeseen (and unforeseeable) situations” (Barnett, 2009, p. 439). Training of the kind described by Barnett is very widespread today and, according to Urdang (2010), it has abandoned both its basic psychodynamic orientation, as well as its emphasis on professional self-construction and on process-oriented work. Instead, training focuses on cognitive-behavioral theories oriented toward finding solutions, and on time-limited and evidence-based treatments that are results-oriented. These approaches tend to reinforce socio-educational workers' tendencies toward “omniscience, benevolence, and omnipotence” (Brightman, 1984–1985). Thus, larger questions related to meaning, inner life, and group dynamics are often overlooked (Applegate, 2004).

Reflexive processes are considered a crucial strategy for socio-educational workers dealing with uncertain, complex, ambiguous, and unpredictable situations (Spafford et al., 2007; Afrouz, 2021). Reflexive processes are also helpful in managing the financial insecurity of the profession (Werzelen et al., 2019). However, a certain amount of uncertainty, in contrast to the centrality of bureaucratic organizations, is equally crucial to this field (Finlay and Gough, 2003; Striano et al., 2018). In this profession, emotions, thoughts, and actions are intrinsically connected. *During* professional activity, it is often a “defended self”: defenses can be exceptionally heightened by difficult situations (Bower, 2005; Trevithick, 2011). For this reason, it is important to cultivate reflection *after* professional activity: such reflection entails a dynamic process of self-awareness that takes into account emotions as well as both external and internal reality.

Although it is not easy to prove the link between reflection, educators' well-being, and the efficacy of interventions to promote team reflexivity, multiple studies have suggested that reflexive practices improve relationships with others and with one's professional practice. It thus protects from the risk of burnout, especially in unpredictable and complex environments (Widmer et al., 2009; Morgan et al., 2013; Matyushkina and Kntemirova, 2019).

Moreover, there are multiple studies on team reflexivity and its positive effects on performance, innovation, and efficacy (De Dreu, 2002; Schippers et al., 2015; Konradt et al., 2016).

The link between reflexivity and specific characteristics of any given team (such as trust, psychological safety, a shared vision, and the diversification of its professional members) has been shown to have a direct, positive effect on the performance of the team (Hinsz et al., 1997). Diverse teams possess a great variety of knowledge, competences, and skills; they also must integrate differing opinions and perspectives on the task at hand. According to van Knippenberg et al. (2004), this process generates more creative and innovative ideas.

The reflexive team is thus an important organizational tool for protecting workers' well-being. Studies have shown that team reflexivity positively affects job engagement (as measured by efficacy, energy, and level of involvement), compassion satisfaction (defined as the pleasure derived from being able to do one's work well while also helping others), and resilience (the ability to respond positively to challenging experiences) (Sanchez-Reilly et al., 2013; Lines et al., 2021).

### The Non-profit Organization “Maestri di Strada”

The non-profit organization Maestri di Strada (MdS, or “Street Teachers”) carries out complex socio-educational interventions, both inside and outside of schools, in order to prevent dropout rates and promote the social inclusion of young people.

Maestri di Strada began in Naples, Italy in 2003 with the “Chance Project,” which offered a second-chance school to young people who had previously dropped out. Since 2010, MdS has launched many new socio-educational projects in partnership with the University of Naples Federico II.

These projects serve the eastern suburbs of Naples: specifically, the San Giovanni, Barra, and Ponticelli neighborhoods. This area has 140,000 inhabitants and covers an area of 20 km^2^. The environment is characterized by high levels of social inequities, poverty, and high rates of early school leaving. Here, criminal organizations recruit many young people who lack both qualifications and employment, and are vulnerable to promises of “quick profits.” Young children below the age of criminal responsibility are often recruited, and the number of young victims of murder is remarkably high. Many of those young people internalize the experience of social exclusion, which transforms into feelings of shame, anger, and a widespread sense of “learned” powerlessness (De Rosa et al., 2017; Parrello, 2018).

Maestri di Strada's interventions follow a theoretical framework which is grounded in cultural psychology and psychoanalysis. Accordingly, all forms of learning and all relationships, whether between clients and workers, or between colleagues, are situated semiotically and characterized emotionally depending on both conscious and subconscious dynamics (Salvatore and Zittoun, 2011).

Maestri di Strada's projects are configured as action research (AR), and often as participatory action research (PAR) (Moreno, 2009; Parrello et al., 2019a).

Action research, as it is known, is a model of investigation whose main aim is to improve the future skills and activities of the researcher, rather than to produce theoretical knowledge. In the 1950s, AR also spread to the field of education (Kaneklin et al., 2010; Baldacci, 2012), and today it is considered one of the most important strategies for qualitative research in education (Coggi and Ricchiardi, 2005), as well as for community care (Lipari and Scaratti, 2014).

At the end of the 1960s, an international network of researchers originated participatory action research (PAR) in order to tackle various problems facing marginalized members of society. Participatory action research deals with power imbalances that generate social and individual distress (Arcidiacono et al., 2017; Stapleton, 2018). For this reason, the PAR method is thought to be particularly suited to the educational field, where power dynamics are a constant presence (Jacobs, 2016). In PAR, knowledge is created by the people involved in the research process, in a non-hierarchical, democratic environment (Savin-Baden and Wimpenny, 2007) that produces contextual knowledge (Pine, 2009).

Reflection is part of the PAR cycle, which can be described as a metacognitive process that consists of exploring personal beliefs, thoughts, and actions in a deliberate, autobiographical, and critical way (Marcosa et al., 2009).

At MdS, both AR and PAR projects aim to identify initiatives that can re-motivate young people to engage with school and to develop active citizenship practices. Young people are assisted individually or in groups by a tutor who is either an adult or a peer a few years older than them. Participants sign up for educational and/or creative workshops, and become actively involved in community events. Moreover, MdS has also developed wider social interventions aimed at supporting families, teachers, and entire communities.

Currently, the MdS team—which seeks to develop multi-disciplinary synergy—is composed of 45 members: educators, teachers, social workers, artists, community parents, psychologists, sociologists, and educational theorists.

Maestri di Strada can be defined as a “community of practices” (Wenger, 1998) because its members are united by a common mission, reciprocal commitment, and a shared narrative repertoire. It is also a “reflective community” (Schön, 1983) because the team's reflexivity is at the center of its methodology (Leitch and Day, 2020). The main reflexive tool used by MdS is the Multi-Vision group (MG) (Parrello et al., 2020).

### The Outbreak of the COVID-19 Pandemic

In March 2020, the World Health Organization declared COVID-19 a pandemic on the basis of its global diffusion. Italy was the first European country to be seriously affected by the virus, and the government promptly implemented very restrictive measures aimed at the entire population. A lockdown was enacted involving all collective economic, cultural, and educational activities, which were substituted, whenever possible, by distance working and learning. All persons were forbidden from leaving home, except in cases of utmost urgency. Such a drastic and sudden form of social distancing and isolation is unprecedented in history.

The spread of the virus, its daily death toll, and social distancing undermined senses of stability, safety, and identity, as well as physical and psychological continuity, for both individuals and communities (van der Kolk, 2014). Thus, the traumatic impact of the pandemic immediately became clear: many experienced immobility, loneliness, uncertainty, a distorted sense of time, the loss of predictability in the world, and a loss of life's meaning.

Various psychological consequences of the pandemic have already been observed: all over the world, mental health services have registered a generalized spread of sleep disorders, anxiety, depression, and post-traumatic stress disorder (Cellini et al., 2020; Galea et al., 2020; González-Sanguino et al., 2020). The mental health of children, teens, and young people is at risk as well: they have been deprived of extra-familial social relationships (Dubey et al., 2020; Ghosh et al., 2020; Orgilés et al., 2020; Parola et al., 2020; Petretto et al., 2020; Rogora and Bizzarri, 2020); they have endured their parents' altered mental states and the increase of intra-familial conflicts (Sommantico, 2010; Fionda, 2020; Usher et al., 2020); and they have been constantly exposed to a terrorizing media narrative, centered around death and disease (Garfin et al., 2020). In addition to psychological consequences, socioeconomic effects must be taken into account as well. Lockdown has often led to unemployment and a rise in poverty rates. One-third of all students in the world (about 8 million in Italy) did not have access to technological devices, a stable Internet connection, and/or adequate home spaces for distance learning. This divide has exacerbated inequalities, the risk of school dropout, and social exclusion: according to UNICEF (2020), this is a “global education emergency,” the impact of which could be felt for decades to come.

Past studies of other severe, unplanned disruptions to schooling and family have shown that the greatest negative impact on long-term educational and emotional outcomes tends to be observed on the most disadvantaged children. Consequently, there are considerable concerns that the acknowledged attainment gap for children and young people from disadvantaged families could be exacerbated by the COVID-19 pandemic (O'Connor et al., 2020).

Given this background, teachers and educators have been exposed to high stress levels, especially those who work in marginalized settings. With the closing of schools and local centers for socio-educational activities, teachers have been forced to abruptly interrupt their relationships with colleagues, students, and families, and to adapt quickly to distance learning—often without adequate training. The demands of the Ministry of Education and of school principals were pressing and at times contradictory. Working hours increased. For these reasons and various others (Parrello et al., 2019b), many researchers have predicted a higher risk of burnout for teachers during the pandemic (Alves et al., 2020; Kim and Asbury, 2020; Sokal et al., 2020; Trust et al., 2020; UNESCO, 2020). The professional difficulties faced by third-sector educators have been similar to those of teachers, with the addition of greater financial insecurity due to their roles' structural precariousness in Italy (De Lauso and De Capite, 2020).

It is clear that the pandemic and social distancing have affected workers' mental well-being, and their performance as teams, both in local schools and with the MdS Association. So how can the well-being of individuals be protected, and how can teams develop resilience in order to face the impact of COVID-19?

Due to health regulations, the MG—the teams' main tool of “thought resistance” —could not be organized in person, in this very moment of utmost uncertainty and anxiety. However, MdS's workers needed to stand together and think together in order to confront the enormous struggles facing local pupils and families, and to recognize their own vulnerabilities (O'Connor et al., 2020; O'Leary and Tsui, 2020; Cabiati and Gómez-Ciriano, 2021).

Thus, aware of the importance of MGs, the MdS workers decided to try to carry them out remotely—a rapidly expanding practice (Barak, 1999; Barnett, 2014; Handke et al., 2019)—in compliance with health regulations.

### The Aim of the Present Study

Through qualitative research with a longitudinal approach, this study aims to evaluate the MG's efficacy in supporting teams' reflexive thinking, despite its new online format during a traumatic event such as the pandemic.

In particular, through a comparison of MG discourse both before and during the COVID-19 lockdown, the study investigates the factors that threaten and protect individual and team well-being, and consequently impact professional performance. The goal was also to evaluate how the pandemic “entered” group discourse, and to investigate if, even in its online format, the MG instrument succeeded in offering an adequate space for confronting such an unsettling and unexpected event, and whether it was able to support resistance, resilience, and creativity.

## Methods

The multivision group (MG) is a modified balint group (Van Roy et al., 2015). In the 1950s, the Balint Group (BG) was conceptualized in order to support the work of doctors. During treatment, doctors can be viewed as the most powerful medicine: as such, after treatment, observation of themselves, the patient, and the doctor-patient relationship is required within the context of a larger group setting (Balint, 1957; Perini, 2013). The BG is considered a learning environment as well as a mediating experience, the goal of which is to improve the following: sensitivity to clients' needs, professional performance, and work satisfaction. Within a BG, a participant presents a case and other members respond with comments, emotional reactions, and hypotheses for alternative practices. The BG is focused less on group dynamics and more collective discussion.

Maestri di Strada's MG is composed of a team of professionals from the organization. They meet on a weekly basis under the guidance of a facilitator, who is also an experienced psychotherapist. Instead of presenting specific cases, participants freely describe whatever work experiences they feel the need to share in that moment, in order to better understand them. The facilitator helps the group to think creatively and to enrich their repertoire of options for handling difficult situations. The goal is to turn individuals' professional problems into shared, collective problems. The atmosphere is focused on listening, on acknowledging emotions as viable pathways to deeper understanding, and on open exchange. The atmosphere is usually open and validating, which allows for more distressing topics to arise as well.

In some ways the MG may resemble the Focus Group (FG): the (FG) is also structured around the way in which participants relate their everyday experience **(**Manuti et al., 2017). However, in the FG, participants do not belong to the same community of practices. Above all, the discussion follows a (more or less) organized track, and is led by a facilitator depending on the level of organized direction desired (Acocella, 2005). In the FG, although the facilitator strives to maintain a calm environment, participants may at times feel strained due to the potential the lack of privacy, and a consequent attempt to avoid difficult topics (Sim and Waterfeld, 2019). Along these same lines, the T-Group (TG) can also at times have damaging effects on its participants, if they do not know one other and are subjected to frequent provocations and to stressful situations (Highhouse, 2002).

All meetings of the MG carried out by MdS took place in the presence of a silent observer (De Rosa, 2003), who then produced a narrative report. Observers are usually trained interns, who come from Social Sciences, Education Science, or Psychology degree programs. They receive brief initial training on observational method from the organization's psychologists. The preferred method is psychoanalytically oriented. While there are no prearranged grids, each person lets their attention drift and focus according to the discourse and dynamics of the group. The narrative report is read aloud during the subsequent meeting. The group then “validates” the report and uses it as a record of its own past history. On the one hand, observers are record-keepers in charge of passively cataloging the universe of meanings present for the group; on the other, however, observers actively contribute to constructing the group archive through their subjective interpretation. Only later on do all group reports become textual material for research.

During the school year 2019–2020, MdS's team met every week for MG meetings at the organization headquarters. Since March 2020, when a health emergency due to the COVID-19 pandemic was declared in Italy and meetings in person were restricted, MdS has carried out weekly *online MGs* through a communication platform (Microsoft Teams). The platform allows participants to listen to speakers and to break in at any time. Only those who speak will automatically appear on screen, with a maximum of nine people at a time. While listening, it is possible to participate in written form through a live chat, which also allows participants to send emojis, images, links, etc. In short, group communication employs three channels: oral, visual (face to face but limited to whoever is speaking in that moment), and written. The facilitator establishes the rules forturn-taking.

Both in person and online meetings were led by an experienced psychotherapist. A silent observer also participated in the meeting, who later produced accurate narrative reports.

This article takes into consideration the MG reports during two different time periods: pre-pandemic (T1) and early pandemic (T2). As a basis of comparison, the school year preceding the pandemic was also taken into consideration. During T1 (September 2018–June 2019), the MdS team met 18 times in person; during T2 (March–June 2020) the team met 12 times through an online platform.

The T1 corpus, composed of 18 reports written by two observers (31.842 occurrences, 5.725 distinct forms, 3.906 lemmas), and the T2 corpus, composed of 12 reports written by two observers (41.173 occurrences, 6.073 distinct forms, 4.094 lemmas), were analyzed by T-Lab Plus software (2020).

T-Lab Plus is an all-in-one set of linguistic, statistical, and graphical tools for text analysis (Lancia, 2004).

Each corpus went through a stemming process (Automatic Lemmatization). A Thematic Analysis of Elementary Contexts was performed. This type of quali-quantitative analysis is suitable for exploring the content of rich narrative and discursive corpora. The starting theoretical hypothesis is that language is, on the one hand, a tool for communicating, to which the principle of relevance and pertinence (e.g., I dwell on a theme because I consider it important) applies. On the other hand, it is also a tool for non-neutral classification, which is indispensable for organizing the world. In the apparent chaos of a conversation (such as the ones taking place during the MG) it is therefore possible to find significant recursions, which are constructed collectively by the group and allow us to penetrate the text in-depth, in order to formulate explicative hypotheses and “local rules” (Smorti, 2003; Giani et al., 2009).

The text was partitioned into elementary context units, with each approximately the length of a sentence. The units were then classified according to the distributions of words in terms of co-occurrences. Cluster analysis was carried out by an unsupervised ascendant hierarchical method (Bisecting K-Means Algorithm). The co-occurrence of semantic features characterizes this analysis. Each cluster consists of a set of keywords (vocabulary) that appear in specific selections of elementary context units, and which were ranked according to the decreasing value of chi-square. A label was then assigned to each cluster by researchers, who first thoroughly read the observation reports that comprise the corpora. This step is fundamental to guaranteeing an interpretive process that limits misunderstandings or simplifications.

Results of the analysis can be considered an isotopic (iso = same; topoi = places) map of the clusters, as they were composed of the co-occurrences of semantic traits. This map can be understood as a picture of the “mind rooms” (or points of view) (Reinert, 1998) in which the MG occurred, and which was subsequently presented to the MdS team in the form of the narrative report.

## Results

### T1—Pre-pandemic Corpus

**Figure 1:**
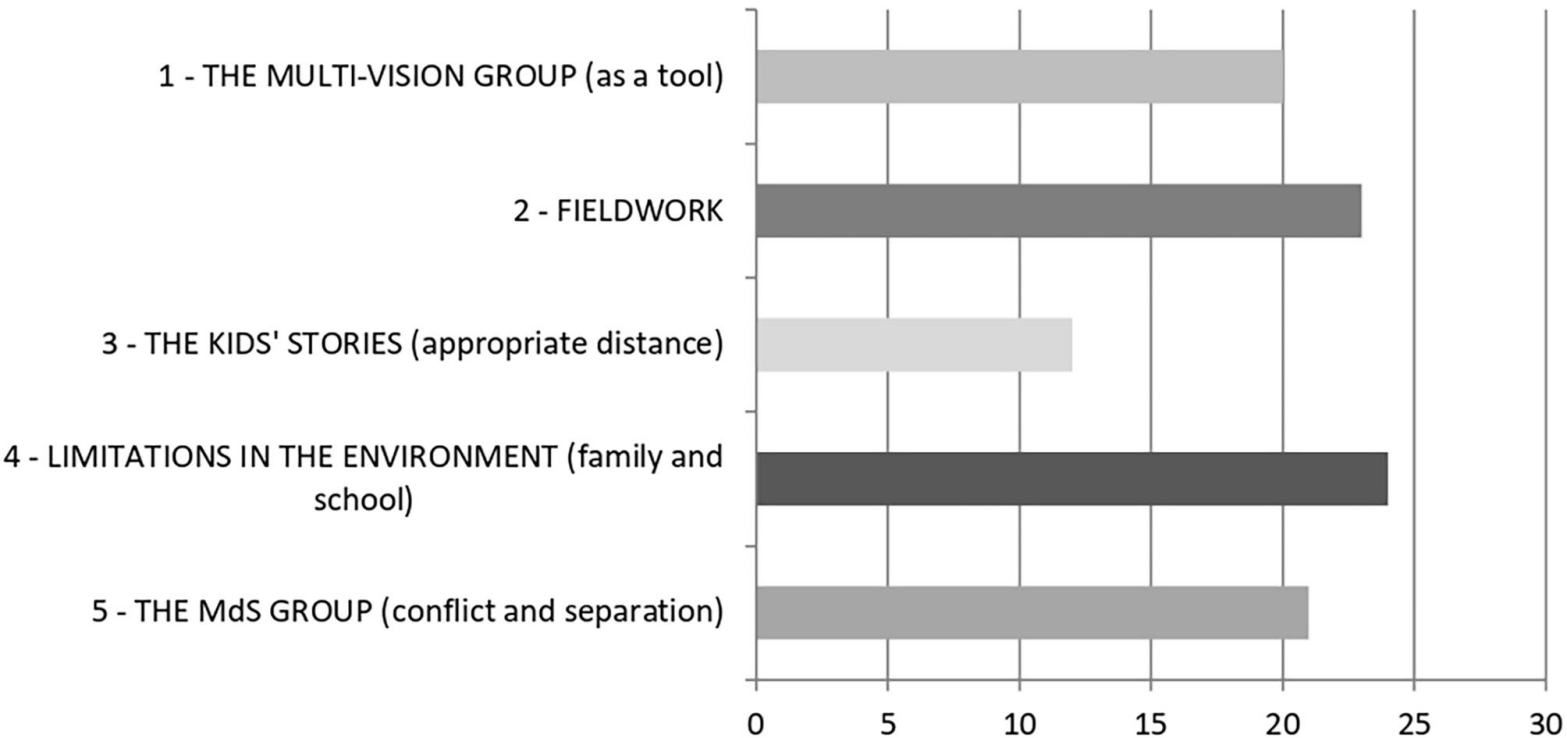
Pre-pandemic Corpus (T1): % of ECUs grouped in each cluster.

The analysis classified 760 elementary context units (ECUs) and partitioned them into five clusters, or macro-themes. Figure 1 shows the clusters' quantitative dimensions. Table 1 displays the specific vocabularies compiled by T-Lab Plus in accordance with the chi-square value.

**Table 1:** Pre-pandemic corpus (T1): the clusters' specific vocabularies.

**Cluster 1**	**CHI2_1**	**Cluster 2**	**CHI2_2**	**Cluster 3**	**CHI2_3**	**Cluster 4**	**CHI2_4**	**Cluster 5**	**CHI2_5**
**The multi-vision group** **(as a tool)**		**Fieldwork**		**The kid's stories:** **appropriate distance**		**Limitations in the environment:** **family and school**		**The MdS group: conflict and separation**	
Multivision	93.926	Job/Work	63.828	Train	42.302	Kid	53.359	Group	47.865
Ilaria (psychol.)	51.347	See	50.554	Ascanio (student)	29.522	Find	48.983	Question	45.099
Antonella (psychol.)	48.757	Week	33.849	Result	24.897	Home	44.065	Cesare (president)	37.974
Start	33.301	Story	28.662	Talk	22.773	Local	31.374	Singles	22.079
Reading	32.475	Salvatore (professor)	26.682	Moment	22.128	Succeed	25.127	Conflict	20.898
Function	29.859	Cira (educ)	24.914	Sara (educ)	21.424	Go out	23.398	Invite (v)	20.468
Saporito	24.360	Community	20.545	Right	21.030	Teach	22.301	Fiorella (educ)	17.335
Meeting	21.293	Change	20.366	Take	16.465	School	20.228	Generate	17.276
Point	19.745	Raffaele (trainee)	20.366	Place	14.856	Theater	18.666	Silvia (educ)	16.126
Carry out	19.602	Educate	18.000	Wheel	14.702	Conditions	18.487	Grow	16.020
Ottavio (trainee)	17.396	Malaise	14.902	Smile	14.702	Create	18.085	Strength	15.359
Peppe (educ.)	17.396	Kid	14.671	Anna (student)	13.321	Feel	16.724	Discourse	15.106
Affirm	16.397	Chiara (educ)	13.800	Suggest	13.321	Simon (educ)	16.724	Grief	14.715
Issue	16.265	Bring	13.378	Close (adj.)	13.179	Sign up	15.679	Mariangela (psychol.)	13.696
Last	16.223	Relationship	13.322	Remember	12.570	Member	15.679	Nicola (educ)	13.482
Return	15.965	Small	13.081	Responsability	11.257	Irvin (educ)	15.580	Occupy	12.492
Last time	15.965	Mds	12.903	Big	10.945	Time	15.266	Problems	12.492
Manner	14.909	Tend to	12.867	Final	10.654	Narrate	15.185	Intervention	12.424
Today	14.909	Allthis	12.867	Organizational	10.654	Save	14.772	Search	12.384
Continuous	13.113	Child	11.791	Dust	10.654	Mother	13.647	Group leader	12.265

*adj., adjective; v, verb*.

### Cluster T1.1—The Multi-Vision Group (20% ECUs)

This cluster's vocabulary contains the team's discourse regarding the MG as a tool (*multi-vision, observation, reading, begin, meeting*) and the *function* of sharing and care that it performs for the group.

Selection of ECUs:

1. After the **reading** of the report, A. (psychologist) notes that during that day's **multi-vision**, its function as a demonstration of care of the group was apparent.
2. (psychologist) recalls that the last **multi-vision** was especially painful and challenging, but members are all present despite the difficulties, “'til desire do us part.”
3. Each member of the group holds a small part of the anguish: this also is the **function** of **multi-vision**.
4. She recalls the anger that emerged from the **last meeting** and says that now we need to face it together.

### Cluster T1.2—Fieldwork (23% ECUs)

In this large cluster, the team reaffirmed the need to pause and step back to *see* both beauty and mistakes in their work, and to reflect on the difficulties of *field work, relationships* between colleagues, and painful encounters with some *kids*. In particular, the team talked about an event organized by the MdS organization, called *Educational Community Week*. They retraced the *story* of the project and the organization of this event, in order to focus on *everything* that did or did not work. They complained about various decisions that were not unanimous. They acknowledged the *malaise* felt by some educators. They questioned themselves regarding what to *change* in the future for the organization of similar events.

Selection of ECUs:

1. Our **job** is dense, full of components that require total immersion. Even when you clock out, you never really get away. But if you don't distance yourself, you can't **see** beauty.
2. What can an educator do in the face of such great pain in a **kid**?
3. C. (MdS' president) highlights an upside of the **Educational Community Week**: the *kids* participated in an event that brought them all together.
4. Some MdS workers experienced the **Educational Community Week** as an imposition, an event that was decided by a few. **Trouble** exploded in the square [where it was held] and did not give them the chance to enjoy the moment.

### Cluster T1.3—The Kids' Stories: Appropriate Distance (12% ECUs)

In this small cluster's vocabulary, the kids' names appear the most frequently (e.g., *Ascanio, Anna*)^1^. Beginning with these stories of marginalization and pain, educators then reflected on their own *responsibility* as adults and professionals, regarding the *right* amount of distance, and how hard it is to obtain certain *results*. They asked themselves when the *right moment* is to *talk, suggest*, or be *close*, but also imagined being able to take a *train* to physically escape this reality, like one of the kids, at least temporarily.

Selection of ECUs:

1. M. (community parent) is worried about **Ascanio**, who recently came out: a fragile kid who feels guilty, isolates himself often and talks of committing suicide. C. (artist) met him on a **train** while he was aimlessly on the run.
2. **Anna** failed and will be held back a year. The problem is not failing per se, but the way it was communicated by the teacher, who was laughing: it was mortifying for her and for us.
3. M. (educator) says that we should maintain the **right** amount of detachment. The GM leader asks: detachment or distance?
4. In this field, people also need to be responsible for stopping when they no longer have the **right** amount of resources or energy to continue, and take care of themselves.

### Cluster T1.4—Limitations in the Environment: Family and School (24% ECUs)

The fourth cluster is very rich: its vocabulary contains references to the shortcomings of family (*home, mother*) and school (*teacher, school*), which were often described in anger, and alongside the desire to *create* the *conditions* necessary to *succeed* in *saving* every *kid*. In particular, the workers talked about MdS's *local* art workshops (*theater*, for example), which were considered to be a valuable resource. However, they also addressed the limits to their own fantasies of omnipotence.

Selection of ECUs:

1. When our kids leave and go **home**, they only find hate; they can't see anything else.
2. How can it be that a deaf-mute 15-year-old **kid** couldn't find anyone at **school** until now who would teach him to communicate, to live in a society, to have a future?
3. If I'm at **school** every morning, I have no **time** to be with them in **theater;** I can't be everywhere, but my heart breaks: tonight, I dreamed of a wall falling on me…
4. If we tell the kids “you'll always find me,” if we think we can “**save**” them, making up for all the shortcomings of their **home** and **school environments**, we then take on a task in which we will inevitably falter.

### Cluster T1.5—The MdS Group: Conflict and Separation (21% ECUs)

In the last cluster, the team dealt with topics concerning internal MdS *group* dynamics. They discussed the feeling of *grief* for the painful exit of an educator (*Fiorella*) from the team, the *problems* regarding democratic processes in the organization, and the *conflicts* between colleagues and the organization's president (*Cesare*). Here, the Group Leader intervened more. They wondered about the well-being of *single* members and about how they could keep *growing* as a *group*.

Selection of ECUs:

1. **F**. (educator) is a symbol for something that all of us are feeling: the fear of failure.
2. S. (educational theorist) says that the group can only work if it is capable of not sweeping the dirt under the rug, and of confronting its own limitations, **conflicts**, and **grief**.
3. We took on a workload that we can only bear with the help of the group: under the weight of the kids' pain, we either completely drown, or we **grow**.
4. C. (MdS' president) encourages us to free ourselves from any sense of dependence on the **boss**.

### T2—Early Pandemic Corpus

**Figure 2:**
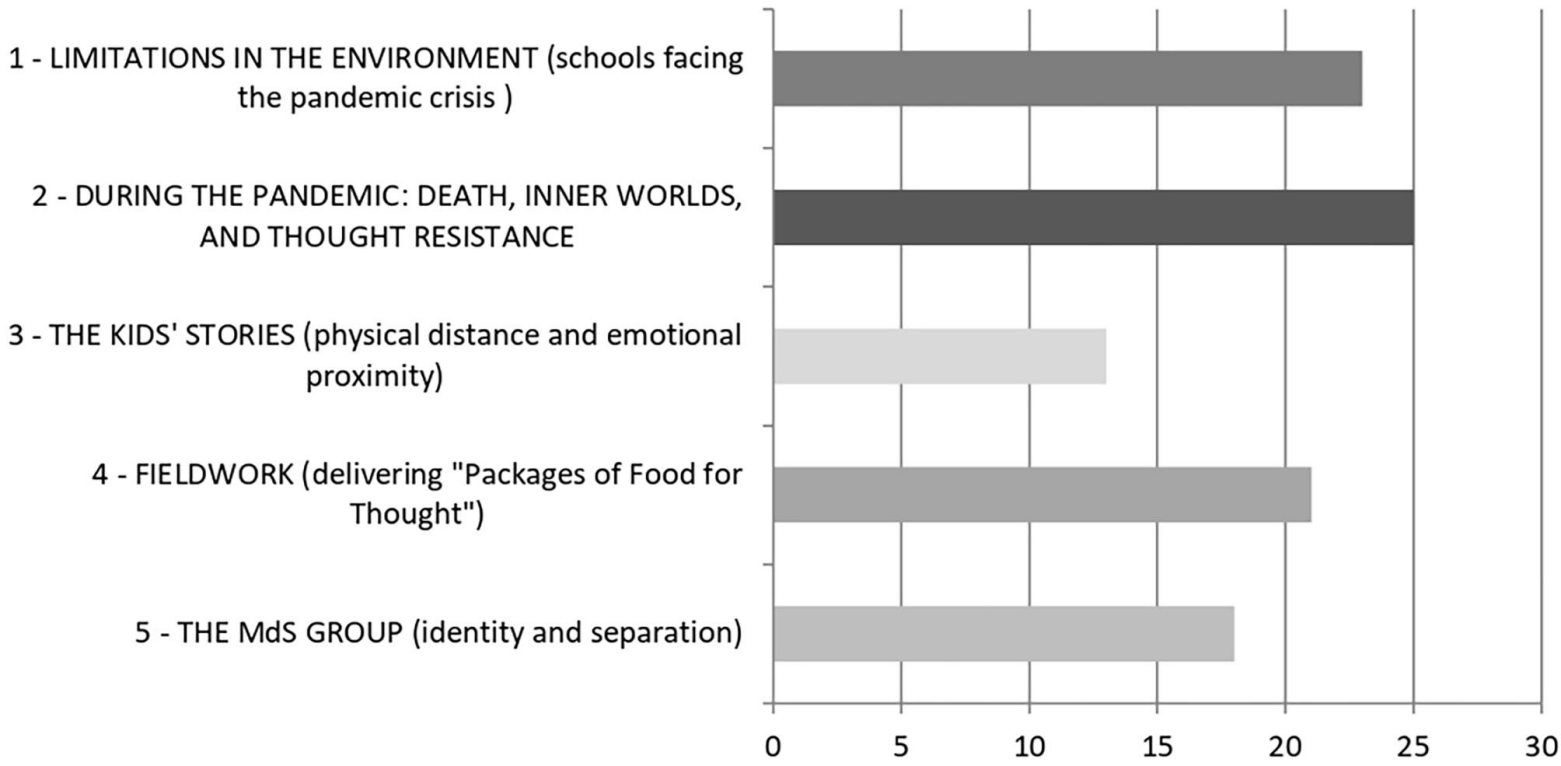
Early pandemic Corpus (T2): % of ECUs grouped in each cluster.

The analysis classified 863 *elementary context units* (ECUs) and separated them into five clusters, or macro-themes. Figure 2 shows the clusters' quantitative dimensions. Table 2 displays the specific vocabularies compiled by T-Lab Plus in accordance with the chi-square value.

**Table 2:** Early pandemic corpus (T2): the clusters' specific vocabularies.

**Cluster 1**	**CHI2_1**	**Cluster 2**	**CHI2_2**	**Cluster 3**	**CHI2_3**	**Cluster 4**	**CHI2_4**	**Cluster 5**	**CHI2_5**
**Limitations in the environment: schools facing the pandemic crisis**		**During the pandemic: death, inner worlds and thought resistance**		**The kids' stories:** **physical distance and emotional proximity**		**Fieldwork:** **delivering “packages of food for thought”**		**The MdS group:** **identity and separation**	
Institution	63.966	Time	48.878	Allow	138.547	Parcel	89.780	Relationship	48.781
Silvia (educ)	55.860	Phase	47.058	Situation	90.218	Mother	75.405	Ask	37.560
School	50.865	Before	39.754	Story	39.032	Place (*n*)	36.640	Pupil	33.208
Power/can	38.548	Return (v)	39.513	(One's) Own	37.253	Eye	34.245	Focus	24.855
System	34.982	Life	36.181	Welcome	32.040	Father	30.335	Distance	24.514
Our	34.701	Normality	31.778	Distance	21.570	Period	30.281	Commitment	21.246
Professional (adj.)	32.099	People	29.057	Ask	18.963	Act	27.545	Didactic	19.544
Formal	28.896	Door	28.501	Write	18.659	Understand	26.437	Bond	17.414
Alone	26.602	Happen	22.979	Development	18.274	Look (*n*)	26.437	Involve	16.948
Context	24.238	War	20.788	Milena (educ)	18.035	See	25.724	Presence	14.704
Social	23.549	Death	19.981	Suffer	16.570	Deliver	22.270	Continuity	13.703
Alternative (adj.)	23.530	Meetings	19.981	Words	16.113	Take	20.841	Limit	13.214
Recognize	23.140	Reflection	19.819	Past	13.739	Delivery	20.571	School	13.000
Add	22.105	Quarantine	17.798	Live	13.338	Love	20.186	Use	12.950
Fields	17.472	Resistance	17.694	Video	12.660	Son/Daughter	18.906	Mds	12.058
Make	17.407	Caracterize	17.204	Ability	12.660	Challenge	18.906	Support	11.696
Role	16.785	Experience	16.925	Narrate	11.527	Health (adj.)	18.787	Online	11.293
Responsability	15.866	Good	16.900	Listen	11.505	Event	18.580	External	10.641
Show	15.753	Allow	16.214	Maintain	10.494	Receive	16.840	Group	10.484
Evaluate	14.838	Moment	15.139	Share	9.964	Narrate	16.574	Day	10.394
Stay	14.571	Live	15.139	Flavio (prof)	8.232	Ornella (educ)	15.393	Enter	10.394
School name	14.571	Mind	14.622	Chiara (educ)	7.008	Move	14.678	Find	10.165
Summer (adj.)	14.505	Feeling	14.329	Talk	6.937	Child	13.281	Network	10.158
Put	14.227	Fear	13.760	Job/Work	6.418	Claudia (educ)	12.968	Function (v)	10.158
Build	14.003	Feel	12.688	Educator	6.347	Teacher	12.932	Emotional	9.630
Territory	12.705	Contagion	12.469	Feel	6.207	Behave	12.932	Contact (v)	9.363
Safe	11.600	Violence	12.248	Energy	6.137	Conclude	12.105	Setting	9.363
Take	11.354	Benecessary	11.986	Powerlessness	6.137	Sara (educ)	11.440	Reflective	9.184
Experiment (v)	10.251	Scare	11.608	Deep	6.073	Adult	10.877	Professor	9.093
Believe	10.216	Full	11.413	Professor	5.982	Immediately	10.877	Parent	8.721
Scholastic	9.511	Hit	10.669	Crisis	5.982	Bring	10.814	Individual	8.096
Force (v)	9.151	Suffering	10.553	Contact (*n*)	5.529	Heart	9.871	Dreams	8.096
Discourse	9.139	New	10.523	Hard	5.449	Street	9.858	Educational	8.028
Teachers	9.050	Multi-vision	10.421	Physical	5.387	Children	8.918	Suggest	7.919
Distance (v)	8.784	Beginning	10.052	Angry	5.213	Start	8.589	Support (*n*)	6.913
Return	8.784	Virus	9.133	Prove	5.213	Workers	8.090	Observe	6.849
Tend to	8.784	Transition	8.987	Spokesperson	5.213	Lotto G (neighborhood name)	8.090	Increase	6.849
Profession	8.621	Act	8.911	Person	5.118	Behavior	8.090	Job	6.849
Reason (v)	8.621	Exit (v)	8.167	Feel	4.982	Remember	7.682	Institutional	6.664
Didactic	8.492	Days	7.816	Suffering (*n*)	4.727	Collective	7.012	Educator	6.528
Job	8.153	Meet	7.816	Narration	4.727	Invitation	7.012	Communication	6.493
Concept	7.924	Present	7.761	Salvatore (prof)	3.986	Gabriele (educ)	6.974	Affective	6.493
Teach	7.254	Narrative (adj.)	7.761	Impossible	3.986	House	6.785	Teacher	6.350
Practice (v)	7.254	Home	7.686	Distance learning	3.986	Understand		Concern (v)	6.043
Dynamic	7.249	Spaces	7.109	Aware	3.986	Irvin (educ)		Possible	5.704
Invite (v)	7.249	Breakage	7.083	–	–	Change (*n*)		Association	5.423
Value	7.249	Powerlessness	6.679	–	–	Field		Colleagues	5.423
Educational	7.092	Concern	6.504	–	–	Wait (*n*)		Process	5.222
School principal	6.631	Lead	6.504	–	–	Touching (adj)		Latest	5.222
Spread	6.631	Necessity	6.432	–	–	Organization		Report (v)	5.193

*adj., adjective; v, verb. n, name*.

### Cluster T2.1—Limitations in the Environment: Schools Facing the Pandemic Crisis (23% ECUs)

This cluster's rich vocabulary contains the group's discourse regarding the functioning of the educational institution in a time of crisis brought about by the pandemic, and in the specific setting of the suburbs (*discourse, institution, school, system, formal, context, social, territory*). The discourse concerns the institution's power and responsibilities (*power, responsibility*). Teachers seem to be alone while facing hardship, stressed by various obligations (*teachers, alone, order (v), practice, work/job, didactic, school principal*): it is harder for them to think things through and experiment (*reason, experiment*) without reflexive tools or group structure. The group wondered how MdS can support an alternative approach to school administration (*can, alternative*).

Selection of ECUs:

1. **Teachers** don't have a group to welcome them in, or a group they can vent to, and they're **alone** within the **institution**.
2. The lack of trust in each other is a characteristic of the **system**.
3. Teachers weren't prepared for **distance learning**, nor for **experimenting** with new **didactic** methods, and now they're forced to by the **school system**.
4. Right now, we need to be understanding of teachers, in order to understand the narrative imposed by the **school system**, but, at the same time, we have to create new **discourses** and **alternative** educational approaches that will be able to support schools now as well as later on.

### Cluster T2.2—During the Pandemic: Death, Inner Worlds, and Thought Resistance (25% ECUs)

This cluster's vocabulary, the largest of the five, contains the group's discourse regarding tragic external events, and their inner reverberations: *time, phase, before, return, normality, quarantine, break, virus, life, death, experience, violence, fear, powerlessness, concern, suffering, war*. They talked about life and death (infection and death rates), about the loss of a sense of normality, and about their new sense of time (denser on the outside and slower on the inside, because of the difficulty of adapting to the rapid succession of events).

Many educators entrusted their inner feelings and strong emotions to the group, often through the use of metaphors and dreams. The main metaphors concerned *war* and disability, while dreams mostly expressed anguish and bewilderment, but also, on occasion, freedom. Many participants explicitly highlighted the beneficial function of the MG: *meetings, reflection, resistance, multi-vision, mind*. Others took notice of positive events occurring at the time: *happen, moment, good*.

Selection of ECUs:

1. The subject of **time** returns: the alteration of time that we have experienced… over the past months, we were forced to change and approach time differently.
2. There is a parallel between this moment in our **lives** and the **experience** of **war**: apprehension, loneliness, constant **fear**, and an inability to take control of our own experience.
3. **Quarantine** feels like **time has** frozen. The illusion of going back to things as they were **before** is insane. But **good** things do **happen, and** are happening.
4. We're lucky because we have the stability of the ongoing presence of the **group**. This is a group that's already established, and capable of emotional sensitivity. We have this **group**-mother that welcomes us and all of our **experiences** and emotions.
5. We at MdS have built a social safety net: that is, the **group**. The group gives us a space for processing, where we can share and think in new ways. So we then find the courage to act responsibly, no matter the difficulties.

### Cluster T2.3—Kids' Stories: Physical Distance and Emotional Proximity (13% ECUs)

This cluster's vocabulary contains discourse regarding the technology that allows people to stay connected despite physical distance (*allow, situation video, distance, story, welcome, contact, share*). However, this type of connection can only develop into emotional closeness and authentic communication if there is also a sense of continuity with a preexisting, positive relationship established by the educator or teacher (*past, maintain, work, educator, teacher*). In that vein, the group recounted stories of violence, powerlessness, and suffering, as told to them remotely by the kids (*spokesperson, story, narration, powerlessness, suffer, suffering*). Some educators thought that the “comfortable distance” itself allowed people to recount those painful experiences.

Selection of ECUs:

1. N. (artist) says that today we can lean on this group for support because positive relationships have already been established before. And he sees the same thing with his students: he can carry out impactful creative workshops from a **distance** because they have shared good experiences together in the **past**. The confidence they find today is rooted in yesterday's positive experience. The kids don't lose the intimacy previously found in the relationship because of the distance; they are comfortable when it comes to continuing to **narrate** their own stories and lives, and through these **stories** we can stay in touch with what is going on for them. It's a sort of virtuous circle that supports our educational work.
2. What if it was this very **distance** that allowed the girl to tell her **story**, to put into **words** so much violence and suffering?
3. This time period makes it very hard to “do,” but it provides an opportunity to listen and think **deeply**.

### Cluster T2.4—Fieldwork: Delivering “Packages of Food for Thought” (21% ECUs)

This cluster's rich vocabulary concerns an initiative set up by MdS during *lockdown*. After initial inactivity due to strict health regulations, MdS introduced the delivery of “packages of food for thought” to kids who belong to vulnerable families (*package, organization, conclude, deliver, delivery, event, health*). The packages contained tablets, books, stationery, and, above all, personal letters. The deliveries took place thanks to the support of the Ministry of Education and law enforcement, and kids and parents saw them as an act of love (*mother, father, son, child, act, love, eye, look, see, touching*). The group discussed the emotional impact of this project at length. Very few teachers wanted to participate (*teacher*). Some of the places they visited were on the outskirts of the suburbs, which are striking in their poverty and neglect (*place, street, house*).

According to the educators, the packages—delivered during a time of collective hopelessness and passivity—kindled a sense of empowerment in the recipients.

Selection of ECUs:

1. **Delivering** a **package** seems like a trivial thing, but it can actually symbolize an act of overcoming certain predetermined limits.
2. The infrequency of **acts** of care was apparent from looking into the eyes of the families. However, the moment they understood who we were, they introduced themselves with a smile and took the **package** and, with it, accepted the **gesture of love**. Like that **father**…
3. The positive thing about delivering **packages** is the effect they created, and the feeling of consideration. People were asking to be **seen**, and they needed attention.
4. They were waiting for us; rumors moved faster than our van. When we arrived in a new location, they were already prepared: initially a little shy and bashful, then curious and eager to receive a **package**, which was viewed as a sign that the organization thought of them, **sees** them, and doesn't abandon them alone in those forgotten, anonymous **places**.
5. Some, curious, got closer and offered their help in navigating the neglected neighborhood **places**.
6. The **teachers** behaved in an ambivalent manner, as if they were torn between seeing the kids and protecting themselves.
7. We had the courage and the ability to **move** in order to reach them. And this was perceived by families as an **invitation** to act, to participate, to exist, and to be **seen** by welcoming eyes that sought their gaze and offered them care.

### Cluster T2.5—The MdS Group: Identity and Separation (18% ECUs)

This cluster's vocabulary records the group's discourse regarding the specific organization of MdS, especially during this time of crisis: their main goal is to cultivate relationships with pupils and parents despite remote learning. They also seek to allow themselves to be “used” by the school and its teachers to serve the students (*relationships, bond, ask, pupil, focus, teachers, parents*), and they continue to dream of a better future for everyone. Again, we find their awareness of having a powerful reflexive tool at their disposal, even when it is only available online (*group, setting, reflexive, support, online, function*). Its absence is regarded as a great limitation for schools (*limit, school, involve*). Selection of ECUs:

1. MdS interposes itself between **teachers**, **pupils**, and **parents** in order to mediate.
2. O. (educator) says that she consciously let the school **use** her to help the **pupils**.
3. One **mother**, worried about her daughter who struggled with distance learning, contacts MdS. O. (educator) gets to work to try and improve communication with **teachers**.
4. Our duty now is to try and protect **relationships**.
5. **Parents** and **children** feel abandoned by the school, and they seek us out, knowing that we are **distinct** from the **school**.
6. **Teachers use** our educators and psychologists in order to vent. They **use** MdS as a container for their **suffering**: indeed, thanks to the **group**, we have learned to contain and withstand the challenges.
7. Without a **reflexive setting** or a welcoming community, **teachers** are scuba divers without oxygen. In the face of this absence, MdS can be a resource: we can be their oxygen!
8. The fact that G. (educator) left the MdS team deeply impacted the **group**. Many wonder if, sooner or later, they too will be forced to leave this job—full of **dreams** but with no financial security.

## Discussion

In July 2020, after 4 months of the pandemic, global research groups began to prioritize education and the need for changes in work environments (O'Connor et al., 2020). The experience of the MdS team is a confirmation of this need.

In the suburbs of Naples, many children and teens live in a state of deprivation and social exclusion: the COVID-19 lockdown increased their risk for school dropout and other challenges^2^ In the same time period, educators' personal and professional well-being was also severely put to the test. The MdS organization attempted to “take care of those who care,” following the suggestions of van der Kolk (2020): by establishing routines, creating thoughtful connection, and fostering the feeling of being alive. The online MG also moved in that direction: it became a regular and eagerly anticipated meeting for members. It allowed participants locked inside their homes to see one other, to communicate, and to re-imagine creative approaches to pedagogy together. The thematic areas that emerged from our analysis of the group's discourses indicate that this reflexive tool was effective: the pandemic took over the group's discussions, and many felt the need to talk about themselves even before they discussed their work, addressing their own vulnerability even before turning to the vulnerability of the kids and their families (Cabiati and Gómez-Ciriano, 2021). In socio-educational work, personal self and professional self are indeed tightly connected (Fellenz, 2016).

Throughout the year prior, during MG meetings, the group had reflected in particular on limitations in the environment, both of families and schools (cluster T1.4, the largest). They also discussed fieldwork (cluster T1.2). They wondered how educators can place themselves at a “right distance” from the kids, especially when their stories are particularly painful and moving (cluster T1.3). The group had also discussed its own internal dynamics, including conflicts between colleagues and with their leader, while reflecting on the fact that one educator had left the team, perhaps because of the difficult emotional sustainability of the job (cluster T1.5). They often mentioned the positive sharing and support provided by the MG tool (cluster T1.1).

Analysis of MG discourse during lockdown revealed four interesting elements. Firstly, the MG allowed participants to face the shock of the pandemic. They made contact with their own and with their colleagues' inner worlds (cluster T2.2, the largest), by expressing emotions and feelings. They also drew from symbolic thinking through the use of metaphors and the recounting of dreams. Thanks to the online MG, educators constructed a shared narrative of the pandemic that differed from the apocalyptic narrative spread by mass media (Rossolatos, 2020), thereby connecting emotions, thoughts, and action, and protecting their own sense of safety, identity, and continuity. The use of metaphors and the critical discussion that ensued (Craig, 2020; Sabucedo et al., 2020), as well as the recounting of personal dreams filled with anguish as well as hope (Iorio et al., 2020), are all signs of an attempt to make sense of the pandemic. Only by taking care of themselves can educators offer protection and support to those they take care of (Myers et al., 2020).

Secondly, the MG group allowed the team to analyze shortcomings in their own professional settings, focusing on the administration of educational institutions facing the pandemic crisis, in order to come up with ways to support the teachers' work (cluster T2.1). The painful stories of the kids “got to them” despite the physical distance, thanks to the relationships that had been consolidated over time (cluster T2.3): a new form of “right distance” was thus experienced through a virtual filter.

Thirdly, a new type of fieldwork emerged: the MdS team promptly came up with creative initiatives, like the delivery of “packages of food for thought” (cluster T2.4), which were useful (and not only materially) to hundreds of struggling students and families.

Finally, the group did not cease reflecting on its own internal dynamics: however, in this phase conflicts seemed to temporarily disappear, perhaps because of a need for internal unity in the face of the collective threat of the virus. Team members then reflected on their professional identity, and on their strengths and weaknesses in supporting school-family relationships and the dream of a fairer society. They were very upset by the fact that an educator left the group because of the profession's financial insecurity (cluster T2.5). During this phase, no comments were made by the team concerning the MG's online format. Reflexive methodology—at first in person, and then online during lockdown—most likely contributed to the resilient and resistant qualities of the MdS community. It was capable of absorbing the shock of a stressful event and undertaking a speedy recovery process. Because of this, perhaps, any tendencies toward depression and self-idealizing hyperactivity were also curbed (Kulig, 2000).

In conclusion, even in the online format it took during the pandemic, team reflexivity helped to achieve many different positive outcomes. It allowed team members to take care of themselves and tend to their own vulnerability (Cabiati and Gómez-Ciriano, 2021), and it had positive effects on performance, innovation, and efficacy (De Dreu, 2002; Fook, 2013a,b; Schippers et al., 2013, 2015). The “packages of food for thought” were the first initiative of Project REM (Educational Resistance in Movement). This Project included, among other programs, online educational art workshops for students, online support groups for parents, online seminars for teachers, and Facebook live streams for all. Each initiative was both informative and entertaining (Parrello and Moreno, 2021). At the end of the school year, all at-risk students who had been served by MdS passed their exams. Cooperation with families increased, and new partnerships with schools were born.

According to O'Leary and Tsui (2020), the pandemic is an invaluable occasion for reflecting, analyzing, and learning from crisis. One can take the time to understand what has been working well and what has not, and identify new ways of working and adapting that can help to further develop the profession. The pandemic and its effects will be felt for many years to come, but it is already clear that socio-educational work is “fundamental to not only rebuilding but transforming our world” (Truell, 2020, p. 548).

The MdS team went back to in-person meetings as soon as it became possible, in early July 2020, with the accompanying awareness that bodies, non-verbal communication, and physical proximity are also fundamental to a group.

## Limits and Future Perspectives

A limitation of this study is that it refers to a single source of observation. In fact, observers' reports are inevitably affected by their subjective experience as trainees They are also sometimes aspiring MdS members. Therefore, we should take into account the possibility of some “complacency” in their perspectives regarding the organization. Further research should integrate this study with questionnaires and interviews in order to also consider the point of view of individual participants regarding in-person, online, or blended MGs (Lodder et al., 2020). A research study integrating observational data and data from a self-report questionnaire evaluating the MG is already under way.

The hope of the authors of this article is that online and in-person reflexive groups may be used more widely by educators inside of schools and in the third sector (Kerkhoff, 2020). New studies should be conducted, offering critical opportunities for comparison.

At the moment, these results cannot be generalized, but the practical implications for workers' well-being appear to be evident.

## Data Availability Statement

The raw data supporting the conclusions of this article will be made available by the authors, without undue reservation.

## Ethics Statement

The studies involving human participants were reviewed and approved by CERP (Comitato Etico delle Ricerca Psicologica), Department of Humanities, University of Naples Federico II. The patients/participants provided their written informed consent to participate in this study.

## Author Contributions

All authors contributed in the same way to conception and design of the study, manuscript composition, and data interpretation. All authors read and approved the final manuscript.

## Conflict of Interest

The authors declare that the research was conducted in the absence of any commercial or financial relationships that could be construed as a potential conflict of interest.

## Publisher's Note

All claims expressed in this article are solely those of the authors and do not necessarily represent those of their affiliated organizations, or those of the publisher, the editors and the reviewers. Any product that may be evaluated in this article, or claim that may be made by its manufacturer, is not guaranteed or endorsed by the publisher.

## Footnotes

^1^Children's names were changed to respect their privacy.

^2^In a single year of the pandemic, reports of school dropout in Naples have risen 65%. Most reports come from the San Giovanni, Barra, and Ponticelli neighborhoods: https://www.oggiscuola.com/web/2021/04/13/abbandono-scolastico-impennata-di-segnalazioni-cagliari-e-napoli-casi-drammatici/.
